# Prevalence of carbapenem resistance and its potential association with antimicrobial use in humans and animals in rural communities in Vietnam

**DOI:** 10.1093/jacamr/dlac038

**Published:** 2022-04-19

**Authors:** Nguyen Thi Phuong Yen, Nguyen Thi Nhung, Doan Hoang Phu, Nguyen Thi Thuy Dung, Nguyen Thi Bich Van, Bach Tuan Kiet, Vo Be Hien, Mattias Larsson, Linus Olson, James Campbell, Nguyen Pham Nhu Quynh, Pham Thanh Duy, Juan Carrique-Mas

**Affiliations:** 1 Oxford University Clinical Research Unit, Ho Chi Minh City, Vietnam; 2 Faculty of Animal Science and Veterinary Medicine, Nong Lam University, Ho Chi Minh City, Vietnam; 3 Sub-Department of Animal Health and Production, Dong Thap, Vietnam; 4 Department of Global Public Health, Karolinska Institute, Stockholm, Sweden; 5 Centre for Tropical Medicine and Global Health, Oxford University, UK

## Abstract

**Background:**

Vietnam and Southeast Asia are hotspots for antimicrobial resistance; however, little is known on the prevalence of carriage of carbapenem resistance in non-hospitalized humans and in animals. Carbapenem-resistant Enterobacteriaceae (CRE), particularly *Escherichia coli* (CREC) and *Klebsiella pneumoniae* (CRKP) and also *Acinetobacter baumannii* (CRAB) are emerging threats worldwide.

**Methods:**

We investigated healthy humans (*n *= 652), chickens (*n *= 237), ducks (*n *= 150) and pigs (*n *= 143) in 400 small-scale farms in the Mekong Delta of Vietnam. Samples (rectal swabs, faecal swabs) were investigated for carriage of CRE/CRAB and were further characterized phenotypically and genotypically.

**Results:**

In the Mekong Delta of Vietnam, the prevalence of CRE isolates in human rectal swabs was 0.6%, including 4 CREC and 1 CRKP. One pig was infected with CREC (prevalence 0.7%). CRAB was isolated from chickens (*n *= 4) (prevalence 2.1%) and one duck (prevalence 0.7%). CRKP was isolated from a human who was also colonized with CREC. The CRKP strain (ST16), from an 80 year-old person with pneumonia under antimicrobial treatment, genetically clustered with clinical strains isolated in a hospital outbreak in southern Vietnam. The prevalence of CRE was higher among humans that had used antimicrobials within 90 days of the sampling date than those had not (4.2% versus 0.2%) (*P *= 0.005). All CRE/CRAB strains were MDR, although they were susceptible to colistin and neomycin. The carbapenemase genes identified in study strains were *bla*_NDM_ and *bla*_OXA_.

**Conclusions:**

The finding of a CRKP strain clustering with previous hospital outbreak raises concerns about potential transmission of carbapenem-resistant organisms from hospital to community settings or vice-versa.

## Introduction

Carbapenems are β-lactam antimicrobials used for the treatment of infections caused by MDR Gram-negative bacteria.^1^ Currently they are classified by WHO as high priority, critically important antimicrobials;^2^ carbapenem-resistant Enterobacteriaceae (CRE) and *Acinetobacter baumannii* (CRAB) (alongside *Pseudomonas aeruginosa*) are regarded as ‘critical, high priority pathogens’ by WHO.^3^ Globally, the incidence of infections with both types of pathogen has been steadily increasing.^4,5^

CRE infections are now being widely reported in Southeast Asian hospital settings.^6,7^ Data from Vietnamese hospitals have documented that this leads to increased mortality and associated health care costs.^8,9^ Prevalence of infection with CRE among patients correlates with length of hospitalization (from 13% on admission to 89% at day 15).^9^ Laboratory data indicate increased prevalence of carbapenem resistance between 2012 and 2016 among *Escherichia coli* (CREC) (from 6% to 8%) and *Klebsiella pneumoniae* (CRKP) (from 17% to 24%).^10^ Recently, two nosocomial CRKP outbreaks caused by distinct lineages of sequence type (ST) 16 have been reported in Vietnam. CRKP strains from Vietnam are typically MDR, and are resistant to colistin.^11,12^ Studies in Ho Chi Minh City (southern Vietnam) (2010–12) indicated that resistance to carbapenems among *Acinetobacter* spp. from ventilator-associated pneumonia patients was 84%–86%, and these had been steadily increasing since 2000.^13^ In another Vietnam-wide hospital study, the prevalence of carbapenem resistance among *A. baumannii* was seen to increase from 70% to 78% between 2012 and 2016.^14^

Carbapenemase production, often encoded by genes located on plasmids, is the most common carbapenem resistance mechanism among Enterobacteriaceae.^5^ Carbapenemases have been further classified into Class A (KPC); Class B (NDM, IMP, VIM); and Class D (OXA-type).^15^ Previous studies in Vietnam have reported a range of carbapenem resistance genes among CREC (*bla*_KPC_, *bla*_KPC-2_, *bla*_NDM-1_, *bla*_NDM-4_, *bla*_NDM-5_, *bla*_OXA_, *bla*_OXA-48_), CRKP isolates (*bla*_KPC-2_, *bla*_NDM-1_, *bla*_NDM-4_, *bla*_OXA_, *bla*_OXA-48_ and *bla*_VIM_),^6,16^ and CRAB isolates (*bla*_OXA-51_, *bla*_OXA-23_, *bla*_OXA-58_, and *bla*_NDM-1_).^17^

Few studies have investigated carriage of CRE among non-hospitalized human subjects. These were carried out in Spain (prevalence 0.4%);^18^ China (3.6% in children;^19^ 2.3% in the general population)^20^ and Cambodia (1%).^21^ In the latter study, *bla*_OXA-48_ was identified both in *E. coli* and *K. pneumoniae*. In addition, a number of studies have demonstrated CRE in animal reservoirs (domestic, wild, companion) and food.^22^ CRE was not detected among 285 livestock samples (including ruminants, pigs and poultry) investigated in Cambodia.^21^ In contrast, a study in China identified CRE in 10.6% and 3.9% of pigs and chickens, respectively.^20^ Another study from China detected CREC, CRKP and *Enterobacter cloacae* in, respectively, 21.8%, 7.4% and 3.9% of poultry samples along the production chain, with *bla*_NDM_ detected in 33.2% samples.^23^

Studies on healthy populations in Netherlands and the USA have identified carriage of *A. baumannii* in 0.9% (faeces) and 10.4% (hands) individuals, respectively.^24,25^ Studies in Germany and Switzerland identified *A. baumannii* in 2.7%–45.7% of poultry samples investigated (choana and raw meat). However, none was identified as CRAB.^26,27^ Previous studies identified exposure to several classes of antimicrobials as a key explanatory factor for colonization with CRE.^28–30^

In Vietnam, information on the prevalence of carriage of CREC/CRKP/CRAB in healthy livestock and in-contact human communities is limited. Vietnam and Southeast Asia are considered hotspots for antimicrobial usage (AMU)/antimicrobial resistance (AMR).^31^ It is not known to what extent this may affect the colonization with CREC/CRKP/CRAB. This knowledge is essential for effective risk management of carbapenem resistance in the country. Using a One Health approach involving co-sampling of animals and human residents, we aimed to investigate: (1) the prevalence of carriage of carbapenem-resistant bacteria among livestock and in-contact humans in the Mekong Delta (Vietnam) and its potential relationship with antimicrobial use; and (2) the genetic determinants of carbapenem resistance in CREC, CRKP and CRAB in this area.

## Materials and methods

### Ethics

The project was conducted in accordance with the Declaration of Helsinki following institutional standards. The study was granted ethics approval by Oxford University Ethics Committee (OxTREC No. 503-20).

### Sample and data collection

The study was carried out in Dong Thap province, considered to be representative of the Mekong Delta region of Vietnam in terms of human and animal demographics. The province has a census population of 1.6 million of whom 80.9% are classed as urban and a human population density of 494.1 per km^2^ (versus 70.4% rural and a population density of 426.8 per km^2^ in the region as a whole). Poultry and pig farm owners in Dong Thap province were randomly selected from the official farm census held by the veterinary authority (Dong Thap Sub-Department of Animal Health, Production and Aquaculture, SDAH-DT). We aimed to recruit 400 farms using a cluster sampling technique (i.e., based on random selection of 20 out of 141 communes in the province, and an average of 20 farms from each commune). Farms raising poultry (chickens or ducks) in flocks with >20 birds, and/or pigs (>2) were eligible. Farmers that consented to the study were enrolled, and their farms were visited during June and July 2020 by SDAH-DT (to collect animal data and samples) and Dong Thap Center for Disease Control (CDC-DT) staff (human data and samples). Data on AMU were collected using structured questionnaires aimed at the person with primary responsibility for animal husbandry. Farmers were also asked to provide all packages (bottles, sachets, etc.) of any antimicrobial-containing products used by humans or animals over the previous 90 and 7 days, respectively. Rectal swabs were collected from 1–3 consenting individuals living in each household. Pooled faecal samples were collected from each type of food-producing animal (chicken, duck and pig) present in the farm. This was achieved by swabbing with a cotton swab, three visibly fresh droppings from each of the target species. Swab samples were placed in 1 mL of sterile brain heart infusion broth (Oxoid, UK) plus 20% glycerol (Sigma, USA).

### Isolation of carbapenem-resistant bacteria

All swab samples were vortexed thoroughly, then a loop of the corresponding suspension was plated onto Chromagar-carbapenem agar supplemented with meropenem 2 mg/L (Nam Khoa, Vietnam) to screen for non-susceptible *E. coli*, *K. pneumoniae* and *A. baumannii*. The plates were incubated at 35 ± 2°C for 20 h. Up to three suspected *E. coli* (reddish), *K. pneumoniae* (metallic blue) and *A. baumannii* (white) colonies from each sample were confirmed using MALDI-TOF (Bruker, Germany). *E. coli* ATCC 25922 was used as a negative control, two CRKP and CRAB were used as positive controls.

### Antimicrobial susceptibility testing

Phenotypic AST was performed using disc diffusion and Etest (meropenem) methods for *A. baumannii.* For CRE and CRAB isolates VITEK 2 (bioMerieux, France) and Sensititre AST was used (Thermo Fisher Scientific, UK). The AST panel included 42 (CRE) and 26 antimicrobials (CRAB) (belonging to 13 classes and 10 classes, respectively) (Table S1, available as Supplementary data at *JAC-AMR* Online). CLSI breakpoints were used for susceptibility interpretation.^32^*E. coli* ATCC 25922 was used for quality control. In this study, isolates that were intermediately resistant to carbapenems were regarded as resistant.

### WGS

Genomic DNA was extracted from carbapenem-resistant isolates using Wizard genomic DNA extraction kit (Promega, US). Genome library preparation was carried out using Nextera XT library preparation kit and WGS was performed on the HiSeq2500 Illumina platform to generate 100 paired-end reads (Macrogen, Korea).

### Data analyses

The prevalence of CREC, CRKP and CRAB in human/animal samples was compared between those individuals/animals using and not using antimicrobials using Fisher’s exact test. Acquired AMR and virulence genes as well as plasmid replicons were identified using SRST2 v0.2.0^33^ with ARG-ANNOT antimicrobial resistance,^34^ BIGSdb virulence genes (https://bigsdb.web.pasteur.fr) and PlasmidFinder^35^ databases, respectively. We used SRST2 with the corresponding MLST scheme downloaded from PubMLST (https://pubmlst.org/mlst). SRST2 used Bowtie2^36^ to map out raw reads against the reference database and SAMtools v1.3^37^ to identify genes and alleles. We assembled all Illumina reads using the *de novo* assembler Unicycler v0.4.8 with the default settings.^38^ Prokka v.1.5 was used to annotate the assembled contigs.^39^

To investigate the phylogenetics of the *K. pneumoniae* ST16 isolate, we combined its genomic data with that from nine isolates obtained from a previous study in Vietnam.^11^ Raw Illumina reads were mapped to the reference genome MGH78578 (CP000647.1) using RedDog pipeline v1.10b (https://github.com/katholt/RedDog). In brief, RedDog used Bowtie2 v2.2.3 to map raw reads and single nucleotide polymorphisms (SNPs) were identified with SAMTools v1.3. Gubbins v1.4.5 was used to remove recombinant regions from the resulting alignment file; SNPs identified in the recombinant regions were subsequently removed, resulting in a final alignment of 142 SNPs. Randomized Axelerated Maximum Likelihood (RaxML) was used to construct a maximum likelihood (ML) phylogenetic tree using GTR + G model of nucleotide substitution with 100 bootstrap replicates.^40^

Sequence data of all CRE and CRAB isolates described in this study have been deposited in the European Nucleotide Archive (ENA) under study accession number PRJEB49509 (ERS8847840 to ERS8847852).

## Results

### Prevalence of CRE and CRAB in human and animals

A total of 652 human rectal swabs were collected from 381 households (19 subjects did not consent to rectal swabbing). About half (49.9%) human subjects were male, with a median age of 50 years (IQR 39–59). The prevalence of CRE isolates from human rectal swabs was 0.6%, including four CREC and one CRKP; CRAB was not found (Table 1). The prevalence of CRE was higher among individuals who had used antimicrobials in the previous 90 days than among those who had not (4.2% versus 0.2%, Fisher exact test, *P *= 0.005).

**Table 1.** Prevalence of CRE/CRAB among animals and in-contact humans stratified by antimicrobial use

Antimicrobial use/source	No. samples	No. samples positive for CRE/CRAB (%)	Bacterial strains (ST)
Humans	652	4 (0.6)	
Used antimicrobials previous 90 days	71	3 (4.2)	01_EC_H^1^ (405), 03_EC_H (38), 04_EC_H (2705), 06_KP_H^1^ (16)
Not used antimicrobials previous 90 days	581	1 (0.2)	02_EC_H (1638)
Chickens	237	5 (2.1)	
Used antimicrobials previous 7 days	118	1 (0.8)	11_AB_C (762)
Not used antimicrobials previous 7 days	119	4 (3.4)	07_AB_C^1^ (762), 08_AB_C^1^ (NF), 09_AB_C (NF), 10_AB_C (NF), 12_AB_C (NF)
Ducks	150	1 (0.7)	
Used antimicrobials previous 7 days	56	0 (0.0)	–
Not used antimicrobials previous 7 days	94	1 (1.1)	13_AB_D (NF)
Pigs	143	1 (0.7)	
Used antimicrobials previous 7 days	29	0 (0.0)	–
Not used antimicrobials previous 7 days	114	1 (0.9)	05_EC_P (398)

AB, *A. baumannii;* EC, *E. coli*; KP, *K. pneumoniae*; identical superscripts indicate same subject; ST, sequence type; NF, not found.

A total of 530 pooled faecal swabs were collected from animals in 400 households (237 chickens, 143 pigs and 150 ducks). The prevalence of carbapenem-resistant CRE/CRAB in chicken samples (2.1%) was greater than in pig/duck samples (0.7% each). In animals, a total of seven CRAB (six from chickens, one from ducks) and one CREC (from pig) were identified. Among chicken samples, the prevalence of CRAB was higher among flocks not recently treated with antimicrobials (3.4% versus 0.8%). In pigs and ducks, carbapenem-resistant bacteria were only detected in (one each) herd/flock that had not been treated with antimicrobials over the previous 7 days (Table 1).

**Figure 1.**
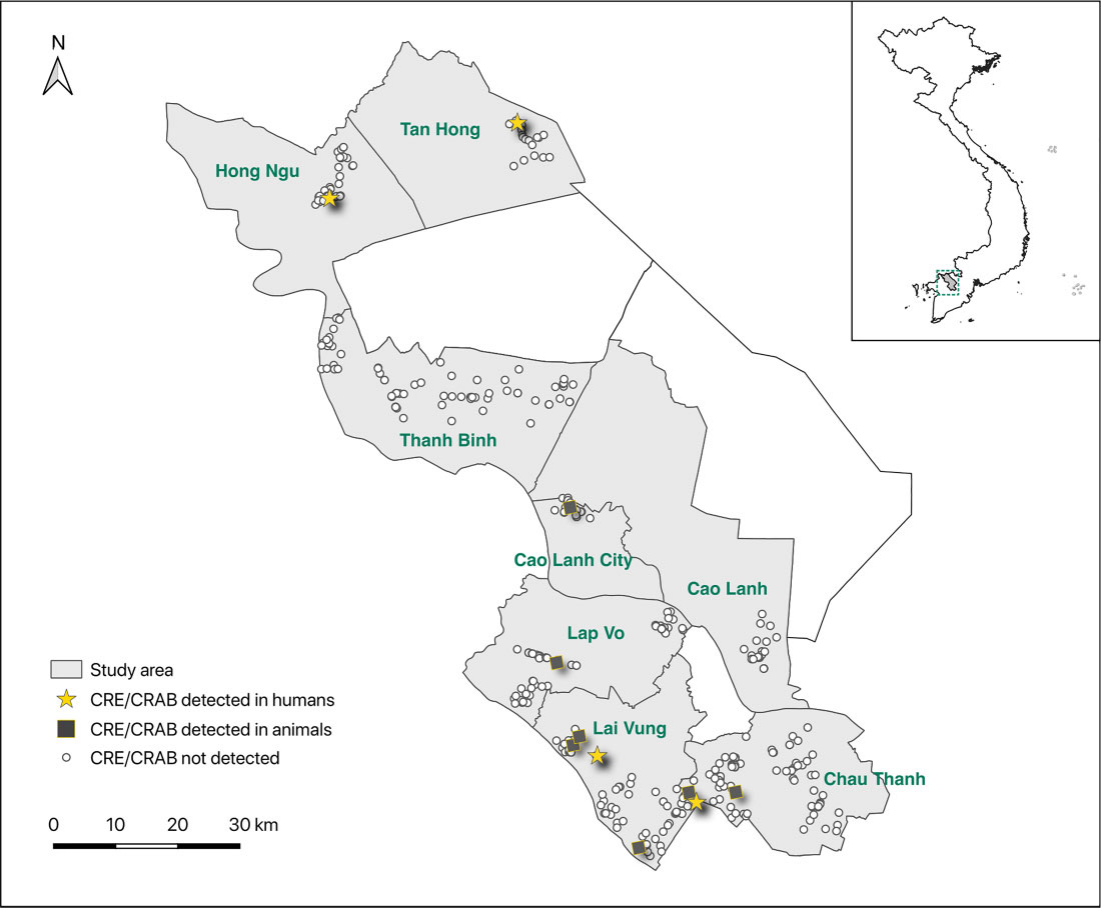
Map showing the collection sites and samples in Dong Thap province.

Most carbapenem-resistant isolates came from different farms and samples, except one CREC and one CRKP isolate from the same human sample, and two distinct CRAB isolates from the same chicken sample by MLST (Table S2). CRE/CRAB bacteria were found in 6 out of 8 districts investigated, but 6/11 (55%) of the CRE/CRAB-positive samples were collected from Lai Vung district (Figure 1) (Tables S3 and S4).

### Antimicrobial consumption and demographic features

Antimicrobial consumption and demographic features of CRE/CRAB-positive hosts are described in Tables S3 and S4. Three out of four human individuals whose rectal swab cultures were positive with CRE had a recent history of AMU within 90 days of the sampling date, including amoxicillin/clavulanic acid (*n *= 2), cefuroxime (*n *= 1), cefixime (*n *= 1), and clarithromycin (*n *= 1). The individual detected with both CREC and CRKP had pneumonia and was being treated with cefixime at the time of sample collection. In one CRAB-positive chicken farm raising fighting cockerels, tilmicosin and gentamicin had been used during the previous week.

### Sequence types (STs)

Based on the MLST profile, we identified five distinct *E. coli* STs: ST2705, ST1638, ST38, ST405 and ST398 (one for each isolate). The *K. pneumoniae* isolate was ST16. Out of the seven *A. baumannii* isolates, two (29%) were identified as ST762, and five (71%) were classified as STNF, (i.e. the ST was not identified based on the current MLST scheme). However, two out of five STNF isolates (from two chickens located on different farms) shared the same MLST profile (Table S2).

### Phenotypic antimicrobial resistance

Among five CREC isolates, ≥80% were resistant to 31/42 antimicrobials (8/13 classes) investigated; the resistance rates were 60% for azithromycin and minocycline, 40% for streptomycin and aztreonam, and 20% for nalidixic acid, ofloxacin, tigecycline and nitrofurantoin. All isolates were susceptible to colistin, amikacin and neomycin. The CRKP isolate was resistant to all antimicrobial agents (39/42) except colistin, neomycin and streptomycin (12/13 classes) (Table 2).

**Table 2.** Summary phenotypic antimicrobial resistance of CREC/CRKP/CRAB isolates

	CREC							CRKP		CRAB								
ID:	01_EC_H^1^	02_EC_H	03_EC_H	04_EC_H	05_EC_P	Human (*n *= 4)	Pig (*n *= 1)	06_KP_H^1^	Human (*n *= 1)	07_AB_C^1^	08_AB_C^1^	09_AB_C	10_AB_C	11_AB_C	12_AB_C	13_AB_D	Chicken (*n *= 6)	Duck (*n *= 1)
Sequence type:	405	1638	38	2705	398			16		762	NF	NF	NF	762	NF	NF		
	MIC (mg/L)					R (isolates)		MIC	R	MIC (mg/L) or zone diameter^a^ (mm)							R (isolates)	
Carbapenem																		
Ertapenem	**≥8**	**≥8**	**≥8**	**≥8**	**≥8**	4	1	**≥8**	1									
Imipenem	**≥16**	**≥16**	**≥16**	0.5	**≥16**	3	1	**2**	1	2	1	1	2	2	**4**	**4**	1	1
Meropenem	**≥16**	**≥16**	**≥16**	**2**	**≥16**	4	1	**≥16**	1	**≥16**	**3**	**3**	**>32**	**≥8**	**≥16**	**>32**	6	1
β-Lactam																		
Cefepime^a^	**≥64**	**16**	**≥64**	≤1	**8**	3	1	**≥64**	1	21	20	20	22	21	21	18	0	0
Cefixime	**≥4**	**≥4**	**≥4**	**≥4**	**≥4**	4	1	**≥4**	1									
Cefotaxime^a^	**≥64**	**≥64**	**≥64**	**4**	**≥64**	4	1	**≥64**	1	**14**	**16**	**13**	**14**	**13**	**14**	**12**	6	1
Cefpodoxime	**>32**	**>32**	**>32**	**>32**	**>32**	4	1	**>32**	1	16	8	16	16	16	8	8	NA	NA
Ceftazidime^a^	**≥64**	**≥64**	**≥64**	4	**≥64**	3	1	**≥64**	1	**14**	**13**	**14**	**14**	**14**	**14**	**13**	6	1
Ceftiofur	**>32**	**>32**	**>32**	**4**	**>32**	4	1	**>32**	1	16	16	16	16	8	16	16	NA	NA
Ceftriaxone^a^	**≥64**	**≥64**	**≥64**	≤1	**≥64**	3	1	**≥64**	1	**17**	**15**	**15**	**16**	**17**	**6**	**15**	6	1
Cefoxitin	**≥64**	**≥64**	**≥64**	**≥64**	**≥64**	4	1	**≥64**	1									
Cefuroxime axetil	**≥64**	**≥64**	**≥64**	**≥64**	**≥64**	4	1	**≥64**	1									
Aztreonam	**≥64**	≤1	**16**	≤1	≤1	2	0	**≥64**	1									
Amoxicillin	**>256**	**>256**	**>256**	**>256**	**>256**	4	1	**>256**	1	128	64	64	128	256	128	256	NA	NA
Ampicillin	**≥32**	**≥32**	**≥32**	**≥32**	**≥32**	4	1	**≥32**	1									
Piperacillin^a^	**≥128**	**≥128**	**≥128**	**≥128**	**≥128**	4	1	**≥128**	1	**15**	**18**	**18**	**17**	**16**	**17**	**14**	6	1
Ticarcillin	**≥128**	**≥128**	**≥128**	**≥128**	**≥128**	4	1	**≥128**	1	**32**	**32**	**32**	**32**	**32**	**32**	**≥128**	6	1
AMC	**≥32**	**≥32**	**≥32**	**≥32**	**≥32**	4	1	**≥32**	1									
TZP	**≥128**	**≥128**	**≥128**	**≥128**	**≥128**	4	1	**≥128**	1	16	8	8	8	8	32	16	0	0
TIC/CLA	**≥128**	**≥128**	**≥128**	**≥128**	**≥128**	4	1	**≥128**	1									
Quinolones/fluoroquinolones																		
Ciprofloxacin^a^	**≥4**	**1**	**0.5**	**0.5**	**0.5**	4	1	**≥4**	1	25	21	23	25	23	23	23	0	0
Enrofloxacin	**>32**	**2**	**0.5**	**0.5**	**0.5**	4	1	**>32**	1	≤0.25	≤0.25	≤0.25	≤0.25	≤0.25	≤0.25	≤0.25	NA	NA
Levofloxacin^a^	**≥8**	**1**	**1**	**1**	**1**	4	1	**≥8**	1	26	25	25	25	25	25	25	0	0
Nalidixic Acid	**≥32**	16	16	4	16	1	0	**≥32**	1									
Ofloxacin	**≥8**	2	2	2	2	1	0	**≥8**	1									
Aminoglycosides																		
Amikacin	4	≤2	≤2	≤2	≤2	0	0	**≥64**	1									
Gentamicin^a^	≤1	**≥16**	**8**	**≥16**	**≥16**	3	1	**≥16**	1	19	19	19	20	21	20	17	0	0
Neomycin	1	1	≤0.5	1	1	0	0	≤0.5	0	≤0.5	≤0.5	≤0.5	2	1	1	1	NA	NA
Streptomycin	16	**>512**	8	**>512**	16	2	0	≤4	0	16	8	≤4	16	32	32	8	NA	NA
Tobramycin	**≥16**	**8**	**8**	**8**	**8**	4	1	**≥16**	1	≤1	≤1	≤1	≤1	≤1	≤1	≤1	0	0
Phenicols																		
Chloramphenicol	**16**	**≥64**	8	**≥64**	**≥64**	3	1	**≥64**	1									
Florfenicol	**32**	**>256**	8	**256**	**256**	3	1	**32**	1	64	64	64	64	64	64	128	NA	NA
Tetracyclines																		
Doxycycline	**16**	**32**	**32**	**16**	**32**	4	1	**32**	1	≤1	≤1	≤1	≤1	≤1	≤1	≤1	0	0
Minocycline	2	**≥16**	**≥16**	4	**≥16**	2	1	**≥16**	1									
Oxytetracycline	**16**	**512**	**512**	**512**	**>512**	4	1	**>512**	1	≤4	≤4	≤4	≤4	≤4	≤4	≤4	NA	NA
Tetracycline^a^	2	**≥16**	**≥16**	**≥16**	**≥16**	3	1	**≥16**	1	21	21	20	19	19	20	19	0	0
Macrolides																		
Azithromycin	**>64**	**>64**	4	**>64**	8	3	0	**>64**	1	2	2	2	4	2	2	4	NA	NA
Sulphonamides																		
Co-trimoxazole^a^	**≥320**	**≥320**	≤20	**≥320**	**≥320**	3	1	**≥320**	1	25	24	24	24	25	25	23	0	0
Trimethoprim	**≥16**	**≥16**	≤0.5	**≥16**	**≥16**	3	1	**≥16**	1									
Nitrofurans																		
Nitrofurantoin	≤16	≤16	32	**64**	≤16	1	0	**256**	1									
Glycylcyclines																		
Tigecycline	≤0.5	**2**	≤0.5	≤0.5	≤0.5	1	0	**4**	1									
Polymyxins																		
Colistin	0.5	0.5	0.5	0.5	≤0.25	0	0	0.5	0	0.5	1	1	1	1	0.5	1	0	0

R, resistant; *n*, number of isolates; NA, breakpoints not available; bold type indicates not susceptible; TIC/CLA, ticarcillin/clavulanic acid; TZP, piperacillin/tazobactam; AMC, amoxicillin/clavulanic acid.

a Disc diffusion method was used for MIC determination.

Seven CRAB isolates were tested for their susceptibility to 26 antimicrobials; however, breakpoints were only available for 17 (Table S1). 100% of the CRAB isolates were not susceptible to meropenem, cefotaxime, ceftazidime, ceftriaxone, piperacillin, and ticarcillin, but fully susceptible to cefepime, colistin, levofloxacin, ciprofloxacin, gentamicin, tobramycin, piperacillin/tazobactam, tetracycline, doxycycline and co-trimoxazole (Table 2). For nine antimicrobials there were no available breakpoints. Overall, the MICs were 16 mg/L for cefpodoxime and ceftiofur (third-generation cephalosporins), ≥64 mg/L for amoxicillin and florfenicol, ≤0.25 mg/L for enrofloxacin, and ≤4 mg/L for oxytetracycline. Variable MICs were observed for azithromycin, neomycin and streptomycin.

### Antimicrobial resistance genetic determinants

**Figure 2.**
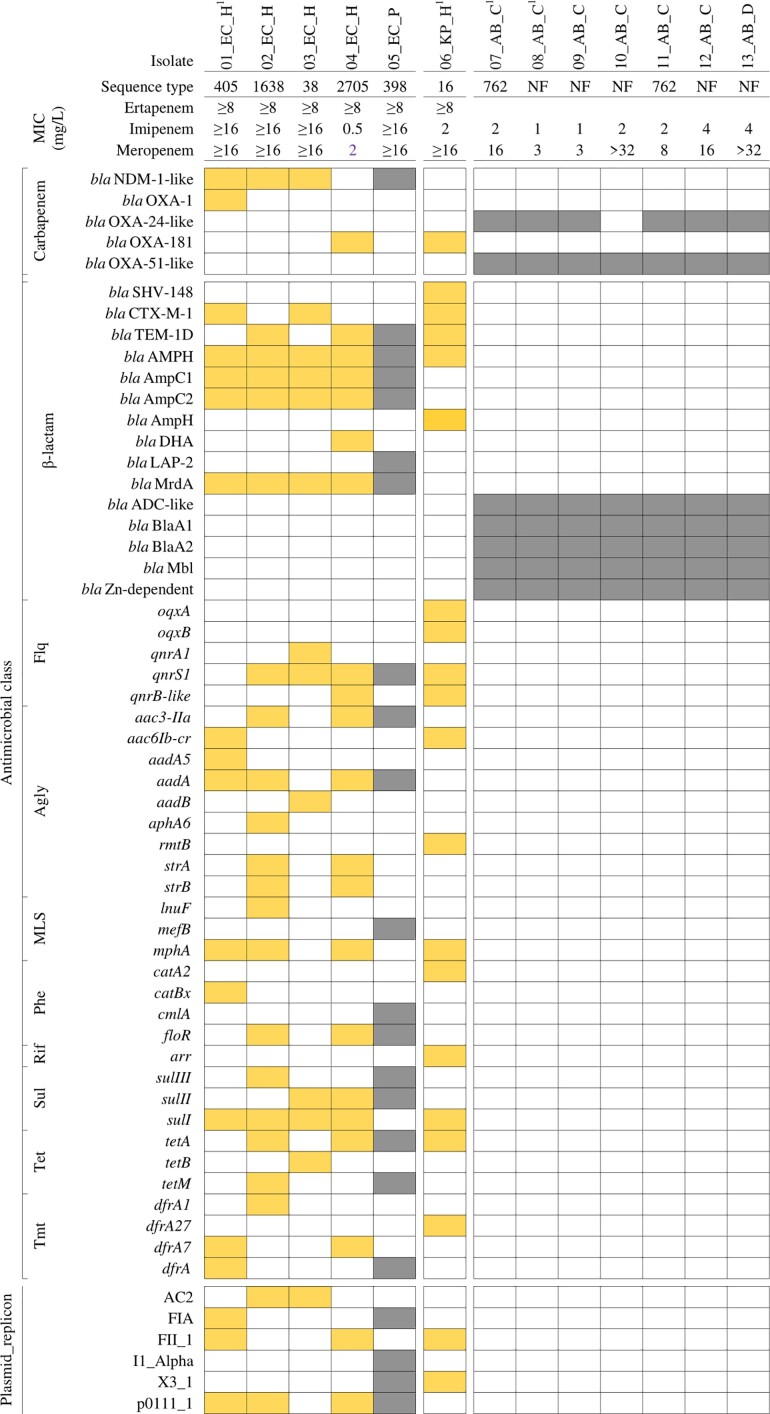
Distribution of carbapenem and other antimicrobial resistance genes in CREC/CRKP/CRAB isolates from humans and animals in Dong Thap province (Vietnam). Colour code: yellow, human isolate; grey, animal isolate. Genes are grouped by encoding resistant to β-lactams, fluoroquinolones (Flq), aminoglycosides (Agly), macrolide/lincosamide/streptogramin (MLS), phenicols (Phe), rifampicin (Rif), sulphonamides (Sul), tetracyclines (Tet), and trimethoprim (Tmt). Isolate species: EC, *E. coli*; KP, *K. pneumoniae*; AB, *A. baumannii*. Subscripts indicate individual samples/subjects. H, human; P, pig; C, chicken; D, duck.

The genotypic AMR characterization of CREC/CRKP/CRAB isolates is displayed in Figure 2. *bla*_NDM-1-like_ genes were found in 4/5 CREC isolates (*bla*_NDM-1_ and *bla*_NDM-5_ were found in 2 isolates each); and *bla*_OXA-181_ in 1/5 isolates. One CREC isolate carried both *bla*_NDM-5_ and *bla*_OXA-1_ genes. β-Lactam resistance genes (*bla*_AmpC1_, *bla*_AmpC2_, *bla*_AMPH_ and *bla*_MrdA_) were detected in all isolates, *qnrS*, *aadA*, and *sulI* were found in four isolates. Genes including *bla*_TEM-1D_ (β-lactam), *aac3-Iid*, *mphA*, *floR*, *sulII* and *tetA* were identified in three isolates.

The *bla*_OXA-181_ gene was detected in the CRKP isolate. CRKP also possessed β-lactam (*bla*_SHV-148_, *bla*_CTX-M-15_, *bla*_TEM-1D_ and *bla*_AmpH_), fluoroquinolones (*qnrS1*, *qnrB-like, oqxAB*), aminoglycosides (*aac6Ib-cr, rmtB*) and other antimicrobial class resistance genes (*mphA*, *catA2*, *arr*, *sulI*, *tetA* and *dfrA27*).

CRAB isolates possessed variants of *bla*_OXA-51-like_ such as *bla*_OXA-75_ (2), *bla*_OXA-208_ (2), *bla*_OXA-70_ (1), *bla*_OXA-91_ (1) and *bla*_OXA-203_ (1), and *bla*_OXA-24-like_ such as *bla*_OXA-72_ (4), *bla*_OXA-143_ (2). The highest MICs of meropenem were seen for strains containing *bla*_OXA-208_ (Table 3). All CRAB strains possessed a similar gene cassette conferring resistance to β-lactams: *bla*_ADC_, *bla*_A1_, *bla*_A2_*, bla*_Mbl_, and *bla*_Zn-dependent_. None of the CREC/CRKP/CRAB isolates harboured *mcr* genes, which confer colistin resistance. Among Enterobacteriaceae, isolates with the NDM types had higher MIC values. For *A. baumannii*, higher MIC values were found for strains with OXA-208 (Table 3).

**Table 3.** Carbapenem resistance-encoding genes and MICs in CREC/CRKP/CRAB isolates

Sample ID	Sequence type (ST)	*bla*_NDM-1-like_	*bla*_OXA-1_	*bla*_OXA-48-like_	*bla*_OXA-51-like_	*bla*_OXA-24-like_	MIC (mg/L)		
							MEM	IPM	ETP
01_EC_H^1^	405	*bla*_NDM-5_	*bla*_OXA-1_				**≥**16	**≥**16	**≥**8
02_EC_H	1638	*bla*_NDM-1_					≥16	≥16	≥8
03_EC_H	38	*bla*_NDM-1_					≥16	≥16	≥8
04_EC_H	2705			*bla*_OXA-181_			2	0.5	≥8
05_EC_P	398	*bla*_NDM-5_					≥16	≥16	≥8
06_KP_H^1^	16			*bla*_OXA-181_			≥16	2	≥8
07_AB_C^1^	762				*bla*_OXA-70_	*bla*_OXA-72_	16	2	NT
08_AB_C^1^	NF				*bla*_OXA-75_	*bla*_OXA-143_	3	1	NT
09_AB_C	NF				*bla*_OXA-75_	*bla*_OXA-143_	3	1	NT
10_AB_C	NF				*bla*_OXA-208_		>32	2	NT
11_AB_C	762				*bla*_OXA-91_	*bla*_OXA-72_	8	2	NT
12_AB_C	NF				*bla*_OXA-203_	*bla*_OXA-72_	16	4	NT
13_AB_D	NF				*bla*_OXA-208_	*bla*_OXA-72_	>32	4	NT

MEM, meropenem; IPM, imipenem; ETP, ertapenem; NT, not tested.

### Phylogenetics of CRKP isolate

**Figure 3.**
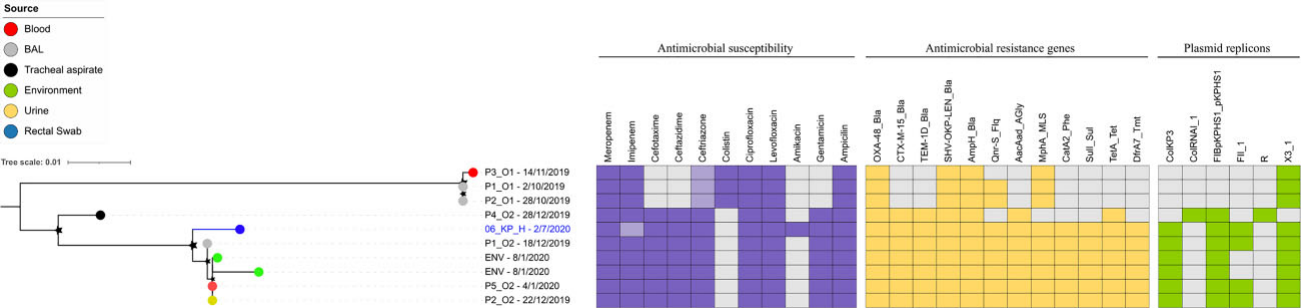
Phylogenetic structure of *K. pneumoniae* ST16 from a human carrier and bloodstream infections.

Our CRKP ST16 isolate clustered tightly with three (of nine) ST16 isolates recovered from patients affected by nosocomial outbreaks at a tertiary hospital in southern Vietnam in December 2019 (Figure 3). The four isolates carried an identical and extensive AMR gene profile including *bla*_OXA-48_, *bla*_SHV_, *bla*_CTX-M-15_, *bla*_AmpH_, *bla*_TEM-1D_, *qnrS*, *aacAad*, *mphA*, *sulI*, *tetA*, and *dfrA7*, predicted to confer resistance to antimicrobials from the carbapenems, cephalosporins, β-lactams, quinolones, aminoglycosides, macrolides, tetracyclines and trimethoprim. Additionally, our CRKP ST16 isolate harboured the identical *bla*_OXA-48_-carrying IncFII plasmid (coverage 90%, identity 99%) with previously identified in outbreak ST16 isolates (accession number: MT635909.1).^11^

## Discussion

This is, to the best of our knowledge, the first report describing carriage of CRE and CRAB in animals and humans living in close contact in rural (Mekong Delta) Vietnam. Our study confirms the presence of CRE in non-hospitalized human subjects and pigs at a relatively low prevalence (0.6%–0.7%). Although we did not find evidence of CRE in poultry species, CRAB was detected in 2.1% and 0.7% chickens and ducks. Carbapenem resistance was encoded by *bla*_NDM-1-like_ (4/5 CREC) and *bla*_OXA-181_ (1/5 CREC and 1/1 CRKP) genes. One CREC contained both *bla*_NDM-1-like_ and *bla*_OXA-1_.

The observed prevalence of carriage of CRE (0.6%) was of similar magnitude to a previous study in rural Cambodia (∼1%); however, in that study CRE was not identified in any of 285 livestock faecal samples. In that study, both CREC and CRKP isolates detected in humans harboured *bla*_OXA-48_.^21^ Previous studies have detected *bla*_OXA-48_-positive *E. coli* in 0.09% (1/1086) and 1.6% (3/183) healthy humans from Switzerland and Lebanon,respectively.^41,42^ In contrast, no CRE was detected in 433 and 320 non-hospital human samples from India or Spain.^43,44^ In China, carriage of CREC was 2.3% of 735 non-hospitalized humans, 3.9% of 305 chickens and 10.6% of 417 pigs (all encoded by *bla*_NDM_ carbapenemase genes).^20^ Another study in Egypt revealed high levels of CRKP carriage in chickens with signs of respiratory diseases (15%) as well as in workers and veterinarians (10% of 49 faecal samples). The CRKP strains carried *bla*_KPC_, *bla*_OXA-48_ and *bla*_NDM_ genes.^41^ A previous study demonstrated a higher prevalence of CRE among Vietnamese hospital patients on day of admission (13%),^9^ a much higher figure than our 0.6% among healthy individuals. It is likely that this difference reflects previous antimicrobial use or exposure to healthcare facilities.

The finding of a CRKP strain in a province identical to that found in a previous nosocomial outbreak in a crowded city in the country^11^ suggests that transmission of CRKP from hospital facilities to the community may occur.

The finding of a CRKP strain in these rural settings identical to that found in a previous nosocomial outbreak in a hospital the country^11^ and the high prevalence of colonization in hospital settings confirms transmission from hospital facilities to the community.

We only detected CRAB in poultry faecal samples, all of which harboured *bla*_OXA-51-like_ genes, and most (6/7) *bla*_OXA-24-like_ genes. A study in Germany identified *bla*_OXA-51-like_-producing *A. baumannii* in choanal swab samples of chickens (2.7%) and geese (7.5%).^26^ CRAB was detected in 5.8% (3/52) and 11.2% (112/1000) poultry meat in Iran and Turkey, respectively.^45,46^ In contrast, a study in Switzerland demonstrated that poultry was the most frequently *A. baumannii*-contaminated type of meat (45.7% of 94 samples); however, none of them were carbapenem resistant.^27^ Meat is suspected to be a potential source of MDR *A. baumannii*, presumably resulting from faecal contamination.^47^ Although we did not investigate meat samples, our study suggests that poultry (including its meat) may potentially be a source of infection of CRAB and therefore this merits further study.

Although none of the four CRE-carrying individuals investigated had been recently treated with carbapenems, we found a strong association between antimicrobial use in the last 90 days and carriage of CRE. A previous study identified antimicrobial usage as the single most important explanatory factor for colonization with CRE.^28^ Further interview data (data not shown) revealed that all CRE-positive individuals had visited health care facilities. It is not known to what extent individuals may have been colonized in these settings. We did not, however, investigate to what extent farming practices and exposure to manure may have contributed to colonization with CRE in human subjects, as shown in the Cambodian study.^21^

Our data was suggestive of geographical clustering for CRAB and CREC, four chicken CRAB and two human CREC isolates came from the same district (Lai Vung).

Our study confirmed the presence of *bla*_NDM-1-like_ and *bla*_OXA_ genes in CREC strains. For meropenem and imipenem, the highest MICs (>16 mg/L) were observed among strains carrying *bla*_NDM-1-like_ genes. We did not, however, find evidence of colistin resistance among any of the tested strains. In addition to colistin, amikacin and neomycin (aminoglycosides) were the three antimicrobials to which all CREC strains were susceptible; in the case of CRKP, only neomycin, streptomycin and colistin had inhibitory activity.

### Conclusions

We demonstrated faecal carriage of *E. coli*, *K. pneumoniae* and *A. baumannii* harbouring carbapenemase genes in humans and animals in the Mekong Delta of Vietnam. The highest prevalence of colonization with CREC corresponded to human subjects previously treated with β-lactams and/or in contact with health care facilities. Our results suggest One Health genomic surveillance for CRE/CRAB to detect potential transmission from hospital settings; this could be implemented by longitudinal follow-up sampling of individuals and their animal contacts after being discharged from hospitals. It would also be important to investigate short- versus long-term fitness of carbapenemase gene-encoding plasmids in these individuals.

## Supplementary Material

dlac038_Supplementary_Data

## Supplementary data

Tables S1 to S4 are available as Supplementary data at *JAC-AMR* Online.

## References

[1] Papp-Wallace KM , Endimiani A, Taracila MA et al Carbapenems: past, present, and future. Antimicrob Agents Chemother 2011; 55: 4943–60. 21859938 10.1128/AAC.00296-11 PMC3195018

[2] WHO . WHO list of critically important antimicrobials for human medicine, 6th revision. 2018. https://www.who.int/publications/i/item/9789241515528.

[3] WHO . Global priority list of antibiotic-resistant bacteria to guide research, discovery, and development of new antibiotics. 2017. https://www.who.int/medicines/publications/WHO-PPL-Short_Summary_25Feb-ET_NM_WHO.pdf.

[4] Higgins PG , Dammhayn C, Hackel M et al Global spread of carbapenem-resistant *Acinetobacter baumannii*. J Antimicrob Chemother 2010; 65: 233–8. 19996144 10.1093/jac/dkp428

[5] Suay-García B , Pérez-Gracia MT. Present and Future of Carbapenem-resistant Enterobacteriaceae (CRE) Infections. Antibiotics (Basel) 2019; 8: 122. 10.3390/antibiotics8030122 PMC6784177 31430964

[6] Malchione MD , Torres LM, Hartley DM et al Carbapenem and colistin resistance in Enterobacteriaceae in Southeast Asia: Review and mapping of emerging and overlapping challenges. Int J Antimicrob Agents 2019; 54: 381–99. 31369812 10.1016/j.ijantimicag.2019.07.019

[7] Peters L , Olson L, Khu DTK et al Multiple antibiotic resistance as a risk factor for mortality and prolonged hospital stay: A cohort study among neonatal intensive care patients with hospital-acquired infections caused by gram-negative bacteria in Vietnam. PLoS One 2019; 14: e0215666. 10.1371/journal.pone.0215666 PMC6505890 31067232

[8] Christoff AP , Sereia AFR, Cruz GNF et al One year cross-sectional study in adult and neonatal intensive care units reveals the bacterial and antimicrobial resistance genes profiles in patients and hospital surfaces. PLoS One 2020; 15: e0234127. 10.1371/journal.pone.0234127 PMC7269242 32492060

[9] Tran DM , Larsson M, Olson L et al High prevalence of colonisation with carbapenem-resistant Enterobacteriaceae among patients admitted to Vietnamese hospitals: Risk factors and burden of disease. J Infect 2019; 79: 115–22. 31125639 10.1016/j.jinf.2019.05.013

[10] Vu TVD , Choisy M, Do TTN et al Antimicrobial susceptibility testing results from 13 hospitals in Viet Nam: VINARES 2016–2017. Antimicrob Resist Infect Control 2021; 10: 78. 33971969 10.1186/s13756-021-00937-4 PMC8112055

[11] Nguyen TNT , Nguyen PLN, Le NTQ et al Emerging carbapenem-resistant *Klebsiella pneumoniae* sequence type 16 causing multiple outbreaks in a tertiary hospital in southern Vietnam. Microb Genom 2021; 7: mgen000519. 10.1099/mgen.0.000519 PMC8190610 33565955

[12] da Silva DM , Faria-Junior C, Nery DR et al Insertion sequences disrupting mgrB in carbapenem-resistant *Klebsiella pneumoniae* strains in Brazil. J Glob Antimicrob Resist 2021; 24: 53–7. 33246210 10.1016/j.jgar.2020.11.003

[13] Nhu NTK , Lan NPH, Campbell JI et al Emergence of carbapenem-resistant *Acinetobacter baumannii* as the major cause of ventilator-associated pneumonia in intensive care unit patients at an infectious disease hospital in southern Vietnam. J Med Microbiol 2014; 63: 1386–94. 25038137 10.1099/jmm.0.076646-0 PMC4170484

[14] Le Minh V , Thi Khanh Nhu N, Vinh Phat V et al In vitro activity of colistin in antimicrobial combination against carbapenem-resistant *Acinetobacter baumannii* isolated from patients with ventilator-associated pneumonia in Vietnam. J Med Microbiol 2015; 64: 1162–9. 26297024 10.1099/jmm.0.000137 PMC4755130

[15] Ambler RP . The structure of β-lactamases. Philos Trans R Soc Lond B Biol Sci 1980; 289: 321–31. 6109327 10.1098/rstb.1980.0049

[16] Berglund B , Hoang NTB, Lundberg L et al Clonal spread of carbapenem-resistant *Klebsiella pneumoniae* among patients at admission and discharge at a Vietnamese neonatal intensive care unit. Antimicrob Resist Infect Control 2021; 10: 162. 34801068 10.1186/s13756-021-01033-3 PMC8606094

[17] Hoang Quoc C , Nguyen Thi Phuong T, Nguyen Duc H et al Carbapenemase genes and multidrug resistance of Acinetobacter Baumannii: A cross sectional study of patients with pneumonia in Southern Vietnam. Antibiotics 2019; 8: 148. 10.3390/antibiotics8030148 PMC6783976 31547482

[18] Gijón D , Curiao T, Baquero F et al Fecal carriage of carbapenemase-producing Enterobacteriaceae: a hidden reservoir in hospitalized and non-hospitalized patients. J Clin Microbiol 2012; 50: 1558–63. 22403422 10.1128/JCM.00020-12 PMC3347124

[19] Pan F , Tian D, Wang B et al Fecal carriage and molecular epidemiology of carbapenem-resistant Enterobacteriaceae from outpatient children in Shanghai. BMC Infect Dis 2019; 19: 678. 31370804 10.1186/s12879-019-4298-3 PMC6670130

[20] Li J , Bi Z, Ma S et al Inter-host Transmission of Carbapenemase-Producing *Escherichia coli* among Humans and Backyard Animals. Environ Health Perspect 2019; 127: 107009. 31642700 10.1289/EHP5251 PMC6910777

[21] Atterby C , Osbjer K, Tepper V et al Carriage of carbapenemase- and extended-spectrum cephalosporinase-producing *Escherichia coli* and *Klebsiella pneumoniae* in humans and livestock in rural Cambodia; gender and age differences and detection of blaOXA-48 in humans. Zoonoses Public Health 2019; 66: 603–17. 31264805 10.1111/zph.12612 PMC6852310

[22] Köck R , Daniels-Haardt I, Becker K et al Carbapenem-resistant Enterobacteriaceae in wildlife, food-producing, and companion animals: a systematic review. Clin Microbiol Infect 2018; 24: 1241–50. 29654871 10.1016/j.cmi.2018.04.004

[23] Wang Y , Zhang R, Li J et al Comprehensive resistome analysis reveals the prevalence of NDM and MCR-1 in Chinese poultry production. Nat Microbiol 2017; 2: 16260. 28165472 10.1038/nmicrobiol.2016.260

[24] Dijkshoorn L , van Aken E, Shunburne L et al Prevalence of *Acinetobacter baumannii* and other Acinetobacter spp. in faecal samples from non-hospitalised individuals. Clin Microbiol Infect 2005; 11: 329–32. 15760432 10.1111/j.1469-0691.2005.01093.x

[25] Zeana C , Larson E, Sahni J et al The epidemiology of multidrug-resistant *Acinetobacter baumannii*: does the community represent a reservoir? Infect Control Hosp Epidemiol 2003; 24: 275–9. 12725357 10.1086/502209

[26] Wilharm G , Skiebe E, Higgins PG et al Relatedness of wildlife and livestock avian isolates of the nosocomial pathogen *Acinetobacter baumannii* to lineages spread in hospitals worldwide. Environ Microbiol 2017; 19: 4349–64. 28925528 10.1111/1462-2920.13931

[27] Lupo A , Vogt D, Seiffert SN et al Antibiotic resistance and phylogenetic characterization of *Acinetobacter baumannii* strains isolated from commercial raw meat in Switzerland. J Food Prot 2014; 77: 1976–81. 25364933 10.4315/0362-028X.JFP-14-073

[28] Marchaim D , Chopra T, Bhargava A et al Recent exposure to antimicrobials and carbapenem-resistant Enterobacteriaceae: the role of antimicrobial stewardship. Infect Control Hosp Epidemiol 2012; 33: 817–30. 22759550 10.1086/666642 PMC4370272

[29] Gasink LB , Edelstein PH, Lautenbach E et al Risk factors and clinical impact of *Klebsiella pneumoniae* carbapenemase-producing *K. pneumoniae*. Infect Control Hosp Epidemiol 2009; 30: 1180–5. 19860564 10.1086/648451 PMC2893218

[30] Falagas ME , Rafailidis PI, Kofteridis D et al Risk factors of carbapenem-resistant *Klebsiella pneumoniae* infections: a matched case–control study. J Antimicrob Chemother 2007; 60: 1124–30. 17884829 10.1093/jac/dkm356

[31] Zellweger RM , Carrique-Mas J, Limmathurotsakul D et al A current perspective on antimicrobial resistance in Southeast Asia. J Antimicrob Chemother 2017; 72: 2963–72. 28961709 10.1093/jac/dkx260 PMC5890732

[32] CLSI . Performance Standards for Antimicrobial Susceptibility Testing: Thirtieth Informational Supplement M100. 2020.

[33] Inouye M , Dashnow H, Raven L-A et al SRST2: Rapid genomic surveillance for public health and hospital microbiology labs. Genome Med 2014; 6: 90. 25422674 10.1186/s13073-014-0090-6 PMC4237778

[34] Gupta SK , Padmanabhan BR, Diene SM et al ARG-ANNOT, a new bioinformatic tool to discover antibiotic resistance genes in bacterial genomes. Antimicrob Agents Chemother 2014; 58: 212–20. 24145532 10.1128/AAC.01310-13 PMC3910750

[35] Carattoli A , Zankari E, García-Fernández A et al In silico detection and typing of plasmids using PlasmidFinder and plasmid multilocus sequence typing. Antimicrob Agents Chemother 2014; 58: 3895–903. 24777092 10.1128/AAC.02412-14 PMC4068535

[36] Langmead B , Salzberg SL. Fast gapped-read alignment with Bowtie 2. Nat Methods 2012; 9: 357–9. 22388286 10.1038/nmeth.1923 PMC3322381

[37] Li H , Handsaker B, Wysoker A et al The sequence alignment/map format and SAMtools. Bioinformatics 2009; 25: 2078–9. 19505943 10.1093/bioinformatics/btp352 PMC2723002

[38] Wick RR , Judd LM, Gorrie CL et al Unicycler: Resolving bacterial genome assemblies from short and long sequencing reads. PLoS Comput Biol 2017; 13: e1005595. 28594827 10.1371/journal.pcbi.1005595 PMC5481147

[39] Seemann T . Prokka: rapid prokaryotic genome annotation. Bioinformatics 2014; 30: 2068–9. 24642063 10.1093/bioinformatics/btu153

[40] Stamatakis A . RAxML version 8: a tool for phylogenetic analysis and post-analysis of large phylogenies. Bioinformatics 2014; 30: 1312–3. 24451623 10.1093/bioinformatics/btu033 PMC3998144

[41] Hamza E , Dorgham SM, Hamza DA. Carbapenemase-producing *Klebsiella pneumoniae* in broiler poultry farming in Egypt. J Glob Antimicrob Resist 2016; 7: 8–10. 27530998 10.1016/j.jgar.2016.06.004

[42] Zurfluh K , Nüesch-Inderbinen MT, Poirel L et al Emergence of *Escherichia coli* producing OXA-48 β-lactamase in the community in Switzerland. Antimicrob Resist Infect Control 2015; 4: 9. 25834728 10.1186/s13756-015-0051-x PMC4381362

[43] Lohiya A , Kant S, Kapil A et al Pattern of antibiotic resistance among community derived isolates of Enterobacteriaceae using urine sample: A study from Northern India. J Clin Diagn Res 2015; 9: LC15-9. 10.7860/JCDR/2015/14230.6254 PMC4572981 26393150

[44] Ríos E , López MC, Rodríguez-Avial I et al Detection of *Escherichia coli* ST131 clonal complex (ST705) and *Klebsiella pneumoniae* ST15 among faecal carriage of extended-spectrum β-lactamase- and carbapenemase-producing Enterobacteriaceae. J Med Microbiol 2017; 66: 169–74. 27902381 10.1099/jmm.0.000399

[45] Askari N , Momtaz H, Tajbakhsh E. Prevalence and phenotypic pattern of antibiotic resistance of *Acinetobacter baumannii* isolated from different types of raw meat samples in Isfahan. Iran. Vet Med Sci 2020; 6: 147–53. 31576672 10.1002/vms3.199 PMC7036315

[46] Kanaan MH G , Al-Shadeedi SMJ, Al-Massody AJ et al Drug resistance and virulence traits of *Acinetobacter baumannii* from Turkey and chicken raw meat. Comp Immunol Microbiol Infect 2020; 70: 101451. 10.1016/j.cimid.2020.101451 32171936

[47] Elbehiry A , Marzouk E, Moussa IM et al *Acinetobacter baumannii* as a community foodborne pathogen: Peptide mass fingerprinting analysis, genotypic of biofilm formation and phenotypic pattern of antimicrobial resistance. Saudi J Biol Sci 2021; 28: 1158–66. 33424412 10.1016/j.sjbs.2020.11.052 PMC7783781

